# Association between Metabolic Syndrome and Premicroalbuminuria among Iranian Women with Polycystic Ovary Syndrome: A Case Control Study: Met Syn. and Premicroalbuminuria in PCOS

**DOI:** 10.5539/gjhs.v5n1p187

**Published:** 2012-11-30

**Authors:** Amir Ziaee, Sonia Oveisi, Azam Ghorbani, Sima Hashemipour, Maryamsadat Mirenayat

**Affiliations:** 1 Metabolic Diseases Research Center, Qazvin University of Medical Sciences, Qazvin, Iran; 2 Department of Community Medicine, Metabolic Diseases Research Center, Qazvin University of Medical Sciences, Qazvin, Iran; 3 Department of Nursing, Metabolic Diseases Research Center, Qazvin University of Medical Sciences, Qazvin, Iran

**Keywords:** polycystic ovary syndrome, metabolic syndrome, premicroalbuminuria

## Abstract

**Objective:**

The aim of this study was to assess the association between premicroalbuminuria and metabolic syndrome in women with Polycystic Ovary Syndrome (PCOS).

**Methods:**

In this case – control study, we analyzed the medical records of 78 women from an endocrinology outpatient center of Booali hospital in Qazvin city in Iran during 2008 to 2010. Anthropometric characteristics, Albumin/Creatinine Ratio (ACR), Lipid profile, Liver enzyme concentration, and occurrence of metabolic syndrome were compared between the two groups. Premicroalbuminuria was defined as ACR>7mg/g.

**Results:**

Mean age of patients with PCOS was 27.2± 2.5 years and mean age of 63 controls was 26.9±2.4 years. Premicroalbuminuria was found in 53.8% of PCOS and 33.3% of control group (p value=0.015). Again, patients with PCOS divided in two groups: ACR>7mg/g and ACR<7mg/g. Higher serum levels of fasting insulin and glucose, blood pressure and more waist circumference were seen in PCOS patients with ACR>7mg/g. Fifty percent of patients with PCOS and ACR>7mg/g fulfilled criteria of metabolic syndrome; whereas no case of metabolic syndrome was found in PCOS patients with ACR<7mg/g.

**Conclusions:**

Premicroalbuminuria is more prevalent in patients with PCOS compared to normal individuals. Metabolic syndrome is more frequently seen in patients with PCOS and premicroalbuminuria against patients with ACR<7mg/g.

## 1. Introduction

Polycystic ovary syndrome (PCOS) is the most common endocrine disorder in adult women (Azziz et al., 2004; Van Houten et al., 2012). About 6-8 percent of all women are diagnosed with PCOS in reproductive age (Patel, Bloomgarden, & Futterweit, 2008). PCOS is associated with insulin resistance attributable to ovarian hyperandrogenism. More than half of women with PCOS are obese or overweight that would predispose them to metabolic disorders including glucose resistance, type II diabetes and cardiovascular diseases (Ehrmann, 2005).

Association between metabolic syndrome and cardiovascular diseases has been confirmed in 3 meta-analyses (Ford, 2005; Galassi, Reynolds, & He, 2006; Gami et al., 2007). It has been reported that metabolic syndrome is also related to microalbuminuria and chronic renal disease (Chen et al., 2004; Zhang et al., 2007).

Prevalence of chronic renal disease in patients with metabolic syndrome was reported as 10% in one study (Kurella, Lo, & Chertow, 2005), compared to 6% in general population. In Annual Meeting of Texas (2009), Pre- microalbuminuria has been defined as a risk of cardiovascular diseases and few studies have investigated the association between PCOS and premicroalbuminuria (Patel, Bloomgarden, & Futterweit, 2008; Duleba, & Ahmed, 2010). It seems to be an associated between of metabolic problems and PCOS which might help in discriminating the risk of future cardiovascular diseases (Caglar et al., 2011).

Because of high prevalence of PCOs in adult females and the relationship between metabolic syndrome, as well as urinary albumin excretion, cardiovascular diseases and type II diabetes, premicroalbuminuria can be regarded as a risk factor for cardiovascular diseases in such patients and their early diagnosis would minimize incidence of cardiovascular diseases in the patients through timely interventions. The aim of this study was to assess the association between premicroalbuminuria and metabolic syndrome in PCOs women.

## 2. Methods

### 2.1 Participants

The present case-control study was conducted on seventy-eight, 20-40 years old, females with PCOS referred to endocrinology outpatient center in Buali hospital Qazvin city in Iran from 21-march-2008 to 21-march-2010. For calculating of sample size of this study, we invited 156 persons (78 persons in each group). The control group was selected from otherwise healthy females with comparable age range who referred to health centers for monitoring of their children. Fifteen females in control group did not accept to follow and 78 PCOS was diagnosed based on NIH scores. Inclusion criteria consisted as age (20-40) with menstrual disorder, and hyperandrogenism who referred to endocrinology outpatient center. Patients with diabetes, cardiac or renal diseases and those taking antihypertensive or antilipid prescriptions were excluded from the study.

Approval was obtained from the ethics committee of Qazvin University of Medical Sciences. All the participants were notified about the goals of the study, the principles of Helsinki Declaration and provided written informed consent.

### 2.2 Definition

ACR was determined on urine samples of each case. ACR greater than 7 mg/g was regarded as premicroalbuminuria.

PCOS was considered as the original 1990 National Institutes of Health criteria (oligoamenorrhea, clinical or biochemical evidences of hyperandrogenism and exclusion of other possible causes).

BMI of the cases was determined after measuring their height and weight.

Metabolic syndrome was defined based on ATP III scores (having at least three of following parameters : waist circumference greater than 88 cm, HDL less than 50 unit, TG equal to or more than 150 unit, FBS equal to or more than 100 mg/dl and Blood Pressure(BP) equal to or more than 130/85 mmHg – (NCEP, 2001).

### 2.3 Measures

Serum insulin level was assayed using ELISA direct method (EIA) supplied by monoband Kits,USA. FBS, Cholesterol, LDL, HDL, Triglyceride, AST (aspartat aminotransferase) and ALT (alanin aminotransferase) were measured using a Hitachi analyzer (Tokyo, Japan).

Albumin was measured by means of immunoturbidometric method (Diasys diagnosis Co). Creatinine was quantified via jaffe kinetic method (Pars Azmoon Co).

ACR was measured by immuno-turbido-metric assay supplied by Pars Azmon Kit made of Iran under license of Germany using Alpha Classic Auto Analyzer.

Serum levels of ALT, AST, and Triglycerides (TG), HDL cholesterol, FBS and fasting insulin were measured for each case. Serum insulin was analyzed by ELIZA method (Monobind co).

### 2.4 Statistical Analysis

Continuous variables were expressed as unadjusted mean values ±SD and compared by using T student test. Categorical data were expressed as frequency and compared by using χ_2_. P value of less than 0.05 was accepted as statistically significant and for some cells of Table 2*2 less than 5, fisher’s exact test was considered. Statistical analyses were performed with SPSS.

**Table 1:** Comparison of Characteristics in case and control groups by T test

Groups Variables	Control (n= 63)µ±SD	Case (n= 78)µ±SD	P-value
Age (year)	26.9±2.4	27.2±2.5	0.498
BMI (Kg/m^2^)	23±0.8	23.9±0.2	0.0001*
FBS (mg/dl)	83±8.6	93.9±8	0.0001*
Fasting Insulin (µIU/ml)	5.2±1.2	10.6±3.9	0.0001*
TG(mg/dl)	119.9±7.5	141.4±16.2	0.0001*
HDL (mg/dl)	51.9±5.4	47.8±7.8	0.0001*
ACR (mg/g)	6.5±1.6	7.5±2.6	0.009*
ALT (IU/Lit)	20.05±2.8	24.8±7.1	0.0001*
AST (IU/Lit)	20.3±2.8	23.4±4.8	0.0001*
SBP (mmHg)	108.9±7.3	116±10.6	0.0001*
DBP(mmHg)	68.2±5.8	71.6±8.1	0.006*
WC(Cm)	82.3±2.7	86.4±5	0.0001*

* P<0.05 considered statistically significant

**Table 2:** Compares of Clinical Profile and Laboratory findings in two groups of PCOS patients with and without premicroalbuminuria

Groups Variables	ACR<7mg/g N=36	ACR>7mg/g N=42	P-value*	χ^2^
ALT > 40(U/L)	0.0	7.1% (n=3)	0.245	2.67
AST > 35(U/L)	0.0	7.1 % (n=3)	0.245	2.67
Fasting insulin > 10 (µ IU/ml)	0.0	69.2 % (n=27)	< 0.001	38.94
FBS ≥ 100 (mg/dl)	0.0	50% (n= 21)	< 0.001	24.63
TG > 150 (mg/dl)	8.3% (n= 3)	50% (n= 21)	< 0.001	15.8
HDL < 50 (mg/dl)	50% (n=18)	71.4 % (n=30)	0.052	3.67
BP ≥ 130/85 (mmHg)	0.0	42.9% (n=18)	0.001	20.1
WC > 88cm	0.0	64.3% (n=27)	< 0.001	35.4

BMI: Body Mass Index, TG: Triglyceride, HDL: High Density Lipoprotein, FBS: Fasting Blood Sugar, BP:Blood Pressure, WC: Waist Circumference.), AST: aspartat aminotransferase and ALT: alanin aminotransferase.

* P<0.05 considered statistically significant by fisher’s exact test

## 3. Results

Seventy-eight patients in study group and 63 cases in control group were assessed. Mean FBS, age, and height were similar in both groups. Mean BMI was less than 30 kg/m^2^ in all cases.

Mean BMI, waist circumference, concentrations of insulin, FBS,TG, HDL,ALT, AST, systolic and diastolic pressure were significantly higher in PCOS patients compared to control group (Table 1).

BMI: Body Mass Index, TG: Triglyceride, HDL: High Density Lipoprotein, FBS: Fasting Blood Sugar, SBP: Systolic Blood Pressure, DBP: Diastolic Blood Pressure, WC: Waist Circumference, AST: aspartat aminotransferase and ALT: alanin aminotransferase.

42 (53.8%) and 21 (33.3%) had ACR more than 7mg/g in PCOS and control group respectively. This difference was significant (χ^2^= 5.93 and p value 0.015).

Components of metabolic syndrome, LFT and insulin level were compared in two groups of PCOS patients with and without premicroalbuminuria (Table 2).

Frequency of metabolic syndrome in women who participated in this study with ACR more than 7mg/g and ACR less than 7mg/g was statistically significant (Table 3) and 15% of patients with PCOS had metabolic syndrome; whereas the metabolic syndrome was not found in patients with ACR less than 7mg/g (p<0.001).

**Table 3:** Chi-square of metabolic syndrome with PCOS and ACR in case and control group

		N	Metabolic syndrome		χ^2^	P-value

			Yes=N(%)	No=N(%)		
Case		78	21(15)	57(40)	19.9	0.001
Control		63	0 (0)	63(45)		

ACR≤7		78	0 (0)	78(55)	30.55	0.001
ACR≤7		63	21(15)	42(30)		
	
PCO	ACR>7	42	21(27)	21(27)	24.63	0.001
N=78	ACR>7	42	21(27)	21(27)		

P<0.05 considered statistically significant

Crude Odds Ratio (OR) for association between premicroalbuminuria and PCOS showed 2.33, CI 95% (1.17-4.64) and p value 0.015, whereas when we have adjusted OR by metabolic syndrome, the results showed that there was not any association between them: OR=1.17, CI 95% (0.55-2.47) p value 0.67. (OR and CI between premicroalbuminuria and PCOS in group with metabolic syndrome).

Therefore metabolic syndrome is a confounder for this association.

## 4. Discussion

The present study have investigated the association between premicroalbuminuria and metabolic syndrome in PCOs women, and the results showed that metabolic syndrome is seen more frequently in patients with PCOS when compared to control group. Insulin level of such patients was significantly more elevated than control group. ACR in patients with PCOS was considerably higher than normal individuals. Additionally patients with PCOS who had premicroalbuminuria more commonly developed metabolic syndrome compared to patients with normal albuminuria.

Findings of current study about higher body weight and insulin resistance in patients with PCOS are concordant with other related reports: In one study, higher prevalence of disturbance in glucose tolerance test in individuals with metabolic syndrome in comparison to persons without this syndrome has been shown (38% vs.19%) (Ehrmann et al., 2006). A research was conducted on patients with PCOS in 2006. Long-term follow up of the patients showed seven-fold higher rate of type II diabetes in them compared to control group (Lo et al., 2006).

In our study mean ACR level in patients with PCOS was significantly higher than mean ACR level in control group. The similar to current study prevalence of patients with ACR more than 7mg/g was significantly higher among PCOS group compared to control group (Patel, Bloomgarden, & Futterweit, 2008), and ACR of patients with PCOS was higher than control group. Sixteen percent of patients with PCOS had microalbuminuria (defined as ACR more than 25 unit) (Ehrmann et al., 2006).

In present study, frequency of metabolic syndrome was higher in patients with PCOS and premicroalbuminuria compared to normoalbuminuric cases that this result is coordinate with the results of another study (30.9% vs. 9%) (Azziz et al., 2004).

It should be noted that ACR levels in patient with metabolic syndrome were higher than normal humans. Regression analysis demonstrated that in persons with ACR more than 15mg/g likelihood of presence of metabolic risk factors for heart diseases is higher than persons with lower ACR (Duleba & Ahmed, 2010), and microalbuminuria is an effective independent risk factor for coronary artery disease and related mortality (Cirillo et al., 2008; Ganie et al., 2012).

In a study on 10096 non-diabetic patients with normal renal function the likelihood of developing chronic renal disease (GFR less than 60ml/min) was compared between individuals with metabolic syndrome and patients without it in 9 years follow up. Probability of developing chronic renal disease in cases with metabolic syndrome was 1.24 times more than persons without it; in view of ensuing diabetes and hypertension during study phase. It was concluded that metabolic syndrome would increase chance of chronic renal disease in non-diabetic persons independently (Kurella, Lo, & Chertow, 2005).

Insulin resistance had been associated with albuminuria (Bianchi et al., 1997; Parvanova et al., 2006). Insulin may affect albumin excretion acutely or chronically.

The limitation of this study was restricting case group to patient diagnosed based on NIH criteria.

## 5. Conclusion

In summary, based on the results of present study, we can conclude that premicroalbuminuria in patients with PCOS compared to normal humans is more prevalent and this sign in women with PCOS is coupled to metabolic syndrome. The incidence of premicroalbuminuria is higher in the presence of metabolic abnormality such as hypertriglycerdemia and low HDL. It is suggestion that longitudinal studies should be designed for evaluating the natural course of premicroalbuminoria in PCO.
